# TRPM7 regulates angiotensin II-induced sinoatrial node fibrosis in sick sinus syndrome rats by mediating Smad signaling

**DOI:** 10.1007/s00380-018-1146-0

**Published:** 2018-03-06

**Authors:** Hongbin Zhong, Tingjun Wang, Guili Lian, Changsheng Xu, Huajun Wang, Liangdi Xie

**Affiliations:** Fujian Hypertension Research Institute, The First Affiliated Hospital of Fujian Medical University, Fuzhou, People’s Republic of China

**Keywords:** Sick sinus syndrome, Sinoatrial node, Angiotensin II, Collagen, TRPM7, Smad2

## Abstract

Sinoatrial node fibrosis is involved in the pathogenesis of sinus sick syndrome (SSS). Transient receptor potential (TRP) subfamily M member 7 (TRPM7) is implicated in cardiac fibrosis. However, the mechanisms underlying the regulation of sinoatrial node (SAN) fibrosis in SSS by TRPM7 remain unknown. The aim of this study was to investigate the role of angiotensin II (Ang II)/TRPM7/Smad pathway in the SAN fibrosis in rats with SSS. The rat SSS model was established with sodium hydroxide pinpoint pressing permeation. Forty-eight rats were randomly divided into six groups: normal control (ctrl), sham operation (sham), postoperative 1-, 2-, 3-, and 4-week SSS, respectively. The tissue explant culture method was used to culture cardiac fibroblasts (CFs) from rat SAN tissues. TRPM7 siRNA or encoding plasmids were used to knock down or overexpress TRPM7. Collagen (Col) distribution in SAN and atria was assessed using PASM–Masson staining. Ang II, Col I, and Col III levels in serum and tissues or in CFs were determined by ELISA. TRPM7, smad2 and p-smad2 levels were evaluated by real-time PCR, and/or western blot and immunohistochemistry. SAN and atria in rats of the SSS groups had more fibers and higher levels of Ang II, Col I and III than the sham rats. Similar findings were obtained for TRPM7 and pSmad2 expression. In vitro, Ang II promoted CFs collagen synthesis in a dose-dependent manner, and potentiated TRPM7 and p-Smad2 expression. TRPM7 depletion inhibited Ang II-induced p-Smad2 expression and collagen synthesis in CFs, whereas increased TRPM7 expression did the opposite. SAN fibrosis is regulated by the Ang II/TRPM7/Smad pathway in SSS, indicating that TRPM7 is a potential target for SAN fibrosis therapy in SSS.

## Introduction

Sick sinus syndrome (SSS), a common clinical arrhythmia accounting for approximately 50% of permanent pacemakers worldwide [1], manifests as bradycardia, sinus arrest, sinoatrial blockage, or bradycardia-tachycardia syndrome [2, 3]. SSS is also an independent risk for severe cardiovascular disorders and mortality [4]. Studies on anatomical morphology of sinoatrial node (SAN) have shown that fibrous tissues in SAN play an important role in the maintenance of normal pacing and conduction of SAN, while the fibrosis of SAN tissues affects the generation of SAN action potential and conduction, resulting in SSS [5–7]. In addition, the atrium in sinus node disease due to extensive atrial fibrosis can lead to an atrial standstill [8]. Mechanistically, abnormal ion channels responsible for the initiation and/or conduction of cardiac action potentials are considered the main electrophysiological mechanism underlying the occurrence of SSS [9, 10]. For instance, increased Ca^2+^ flux promoted the pathogenesis of SSS [11] and the suppression of muscarinic-gated K^+^ channel by chemicals or genetic deletion reduced SSS in a mouse SSS model [12]. In addition, angiotensin II (Ang II) is well documented to promote the formation of cardiac myoblasts (CFs) into cardiac myofibroblasts (CMFs), which is a key process in the pathogenesis of cardiac fibrosis [13] and intracellular Ca^2+^ flux plays an important role in this transformation process [14].

The transient receptor potential (TRP) family is a group of highly conserved genes that encode membrane proteins that act as ion channels including Ca^2+^ and Mg^2+^ ions [15] and is involved in the mediation of a variety of cellular events and in the pathophysiology of numerous human diseases including cardiovascular disorders [16]. The TRP family is currently divided into seven subfamilies, including the transient receptor potential melastatin (TRPM) subfamily [15]. Among this subfamily of 8 members, TRPM7 is localized in both the plasma membrane and intracellular organelles and exhibits both protein kinase activity and ion channel functions [17]. TRPM7 is highly expressed in SAN, and the knockout of TRPM7 in both zebrafish and mice interfered with cardiac automaticity [18], indicating the importance of TRPM7 in SAN homeostasis. In addition, TRPM7 signaling appears to functionally interact with Ang II signaling. For example, Ang II stimulation upregulated TRPM7 expression in vascular smooth muscle cells [19], while TRPM7 regulated the downstream molecular phenotypes of CFs induced by Ang II [20]. In the clinic, TRPM and its mediated Ca^2+^-influx signal in CFs of SSS patients have been shown to play a key role in the transformation of CFs into CMFs [21], which was supported by the findings that TRPM7 contributed to the Ang II-mediated progression of atrial fibrosis through the regulation of influx of Ca^2+^ and Mg^2+^ [22]. It has been well recognized that the Ang II-mediated TGF-β1/Smad pathway plays an important role in promoting CFs to secrete extracellular matrix and in myocardial collagen deposition [23–25]. The molecular basis underlying how TRPM7 is incorporated into Ang II-mediated TGF-β1/Smad signaling to direct the development of SSS has not been well defined, although one study proposed that TRPM7 was potentially required in TGF-β-induced fibrogenesis in human atrial fibrillation [26] and another study showed that TRPM7 mediated TGF-β1-elicited collagen expression in hepatic stellate cells [27].

In the present study, we established a rat SSS model with different degrees of SAN fibrosis and then investigated the changes in the levels of Ang II, TRPM7 and Smad2 in these rats in vivo. We also employed gain- and loss-of-function approaches to further examine the effects of TRPM7 on Smad2 signaling and fibrosis in Ang II-induced transformation of CFs into CMFs in vitro.

## Materials and methods

### Animal

A total of 48 Sprague–Dawley (SD) rats (12-week-old males, weighed 250 ± 10 g) were purchased from the Shanghai SLACCAS Laboratory Animal Co., Ltd (Shanghai, China; Certificate No. 20120005). Four rats were housed per cage with free access to tap water and food. The room was automatically controlled under a constant temperature of 22 ± 2 °C and humidity of 55 ± 5% with a 12-h artificial light/dark cycle. The animals were randomly divided into six groups: normal control (ctrl, *n* = 8), sham operation (sham, *n* = 8), postoperative 1-week SSS (SSS1, *n* = 8), postoperative 2-week SSS (SSS2, *n* = 8), postoperative 3-week SSS (SSS3, *n* = 8), and postoperative 4-week SSS (SSS4, *n* = 8), respectively. All animal experiments were approved by the Animal Ethics Committee of Fujian Medical University in China (No. 2017-069). All surgery was performed under anesthesia with pentobarbital sodium and all efforts were made to minimize animal suffering. If rats were found in the moribund state such as a strong reduction of food or water intake, weight loss (more than 20%), labored breathing, and unconsciousness or unresponsiveness to external stimuli, they were euthanized with carbon dioxide and death was verified by monitoring for cardiac cessation and respiratory arrest.

### Rat SSS model

**Fig. 1:**
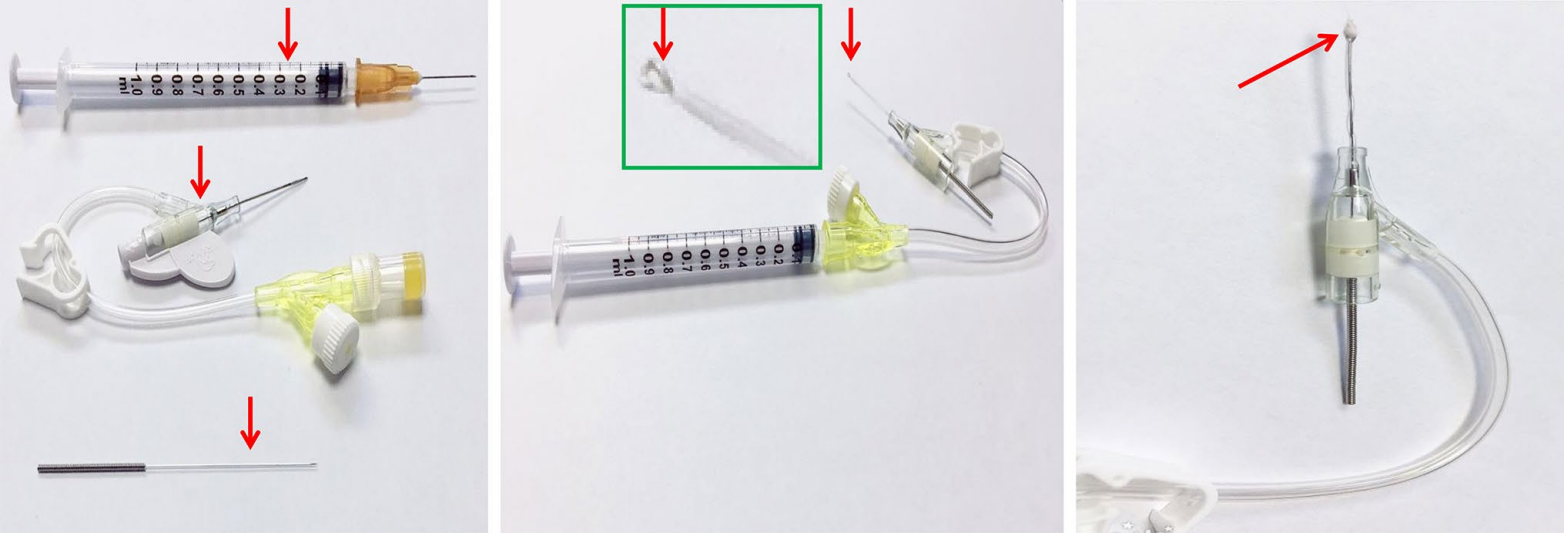
Custom-made device for establishing a rat SSS model. The arrows indicate a syringe with a 1-ml volume, pediatric indwelling needle, an acupuncture needle (left panel), and a circle made at the end of the acupuncture needle (middle panel), where cotton was used with half in the casing pipe and the other half protruding from the casing pipe (right panel)

The SD rats were anesthetized with an intraperitoneal injection of 2% pentobarbital sodium (50 mg/kg bodyweight) for 2–3 min, and then placed on an operation table and connecting to a PowerLab multichannel physiological recording system (AD Instruments, Australia). A tracheostomy was performed to connect the small animal ventilator (BME Co., Ltd, Jiangxi, China) to obtain respiratory support, at a respiratory rate of 60 breaths/min and ventilation volume of 20–25 ml/min. A 1-cm longitudinal incision in the second intercostal space of the right chest of the rat was made to open the skin, the chest muscles were bluntly separated, the second and third ribs were opened using an eyelid speculum device, and the pleura was cut. A sterile cotton ball was used to cover the thymus and right lung tissues, while the right atrial appendage and superior vena cava were exposed. A syringe with a 1-ml volume and pediatric indwelling needle with an approximately 2-mm diameter cotton ball was used (Fig. 1). The small cotton ball was soaked in 20% sodium hydroxide solution in a syringe and then used to perform pinpoint pressing permeation in the rat SAN region at the junction between the right atrial appendage and the superior vena cava for 3–5 min under a steromicroscope (SMZ445, Nikon, Japan). Once the heart rate of the rats decreased by 31–40%, which was monitored by electrocardiogram, the cotton ball was removed. A rat SSS model was considered to be successful if a slow heart rate was stabilized for 2 h [28–30]. The animals in the sham group were treated with normal saline at the same sites for 3–5 min.

### CFs culture

Male SD rats (body weight 250 ± 10 g) were immediately immersed in 75% ethanol for 1–2 min after cervical dislocation, followed by dissection of the thoracic cavity and exposure of the heart for SAN tissue collection. SAN tissues were rinsed 2–3 times in M199 culture medium (Gibco Life Technologies Co., Ltd, Shanghai, China) and sectioned approximately 1 mm^3^ in size. Tissue pieces were transferred to a culture flask with a pipette and evenly placed at the bottom of the flask, and 3–5 ml M199 medium containing 10% fetal bovine serum (Gibco) was then added. The flasks were placed in the incubator at a 45° angle, reversed after 2–3 h, and then placed flat so that the tissue pieces were immersed in the medium. Culture continued until the cells covered two-thirds at the bottom of the culture flask. The cells were digested in 0.25% trypsin and passaged for 3–6 generations for future experiments.

### Transient transfection

CFs at 3–6 generations were digested in 0.25% trypsin, resuspended in M199 medium containing 10% fetal bovine serum, seeded on 6-well culture plate (10^5^ cells/well), and cultured to 60–70% confluency. The medium was then replaced with serum-free M199 culture medium for further incubation for another 24 h. The CFs were transfected with siRNA or encoding vectors of interest using an siRNA transfection kit or plasmid transfection kit (Roche, USA) according to the manufacturers’ instructions. 24 h after transfection, the knockdown efficiency was evaluated using reverse transcription followed by quantitative polymerase chain reaction (RT-qPCR) or western blot.

### PASM–Masson staining

SAN and atrial tissues were fixed in 10% formalin immediately after dissection, paraffin-embedded, and sectioned at 7 µM thickness. Tissue sections were deparaffinized in xylene solution, rehydrated through 100 to 75% gradient alcohol, and eventually rinsed in running water for 10 min. After then, the tissue sections were immersed in 3% fresh periodic acid aqueous solution for 20 min, washed three times in distilled water for 3 min each, and immersed in working hexamine-silver solution to stain at 58–60 °C for 50–60 min. The staining was confirmed under light microscopy, showing an obvious black staining on the reticular fibers. The tissue sections were then stained with 0.2% gold chloride aqueous solution for 1–2 min after 3 washes. Thereafter, the tissue sections were immersed in 0.25% sodium thiosulfate aqueous solution for 2–3 min, and then counter-stained in hematoxylin solution. Finally, the tissue sections were stained in Masson dye for 5–7 min. These stained sections were then observed and imaged under a light microscope Nikon 80i Microscope (Nikon, Japan).

### ELISA

Levels of Ang II, type I and III collagen were detected using ELISA kits (Beijing Zhongshan Golden Bridge Biotechnology Co., Ltd, Beijing, China) accordance to the manufacturer’s instructions and the absorbance at 450 nm was read. The relative Ang II and collagen levels were calculated according to the regression equation of the standard curve calculated by the concentration and the optical density value.

### Immunohistochemistry

Immunohistochemical study was performed as previously described [31]. Briefly, paraffin-embedded sections of SAN tissues were deparaffinized in xylene and rehydrated in an alcohol gradient solutions. Antigen retrieval was carried out in 0.1 M sodium citrate buffer (pH 6.0) at 95–98 °C for 10 min. Then, the tissue sections were washed, blocked for endogenous peroxidase activity, and pre-incubated with goat serum. Subsequently, the sections were incubated with TRPM7 antibody [Abcam] at 4 °C overnight, followed by a 45-min incubation with biotin-labeled rabbit anti-mouse IgG secondary antibody, and the color was developed with a DAB peroxidase substrate kit (Beijing Zhongshan Golden Bridge Biotechnology Co., Ltd). The nuclei were counter-stained with hematoxylin. As negative controls, immunostaining was performed by incubating samples with PBS instead of a primary antibody. The sections were observed under light microscope Nikon 80i Microscope (Nikon, Japan).

### Western blot

Western blot was performed as previously described [32–34]. Briefly, total protein was purified from 50 mg rat SAN tissues or CFs using 1 ml RIPA buffer containing cocktail and 1 mM PSMF (Beyotime Institute of Biotechnology, Jiangsu, China) and separated in 10% SDS-PAGE. The protein was transferred to a semi-dry blotter for 45 min and blocked in 3% skim milk at room temperature for 30 min. The protein blot was then incubated with a primary antibody [p-Smad2 and smad2 (Sigma Co., Ltd, USA); TRPM7 (Abcam Co., Ltd, USA); β-actin (Santa Cruz Biotechnology, CA, USA)] at 4 °C overnight, followed by an incubation with 1:5000 secondary antibody at 37 °C for 1.5 h. The specific protein bands were revealed with an enhanced chemiluminescence detection system (Beyotime Institute of Biotechnology, Jiangsu, China) and quantified using ImageJ (National Institutes of Health) and normalized to the β-actin.

### Total RNA extraction and real-time PCR

Total RNA extraction and real-time PCR study were performed as previously described [32]. Briefly, total RNA was purified from 50 mg rat SAN tissues and CFs using 1 ml Trizol reagent (Life Technology, USA) according to the manufacturer’s instructions. The RNA concentration and purity were determined at A260 nm and A280 nm wavelengths and RNA samples with an A260 nm/A280 nm ratio ranging from 1.8 to 2.0 were used for reverse transcription and cDNA synthesis using a Takara PrimeScript Reverse Transcription Reagent Kit according to the manufacturer’s instructions. The cDNA samples were stored at − 20 °C for future analysis. RT-PCR was performed in accordance with the instructions included with the Takara PrimeScript PCR Reagent Kit, and the primer design and synthesis were performed by Takara Bio, Inc. (Dalian, China). Primer sequences for the RT-PCR are listed as follows: Forward primer of TRPM7: 5′-AGTATATCGTCTGGAGGAGAGTTC-3′; reverse primer of TRPM7: 5′-ATTTGGGTTTCATCTGATTAAAGGC-3′; forward primer of GAPDH: 5′-CTCATGACCACAGTCCATGCCA-3′; and reverse primer of GAPDH: 5′-GCCTTGGCAGCACCAGTGGATG-3′. The target gene expression was calculated relative to the expression of the internal reference gene, GADPH.

### Statistical analysis

SPSS 18.0 (SPSS, Inc., Chicago, IL, USA) software was used for the statistical analysis. The measurement data are presented as the mean ± standard deviation ($\bar{x}$ ± s). One-way ANOVA was used to compare the means among multiple groups. The least significant difference method was used for the pairwise comparison of two means of independent groups. *P *< 0.05 was considered a significant difference.

## Results

### Increased SAN and atrial fibrosis and Ang II levels in SSS rats

**Fig. 2:**
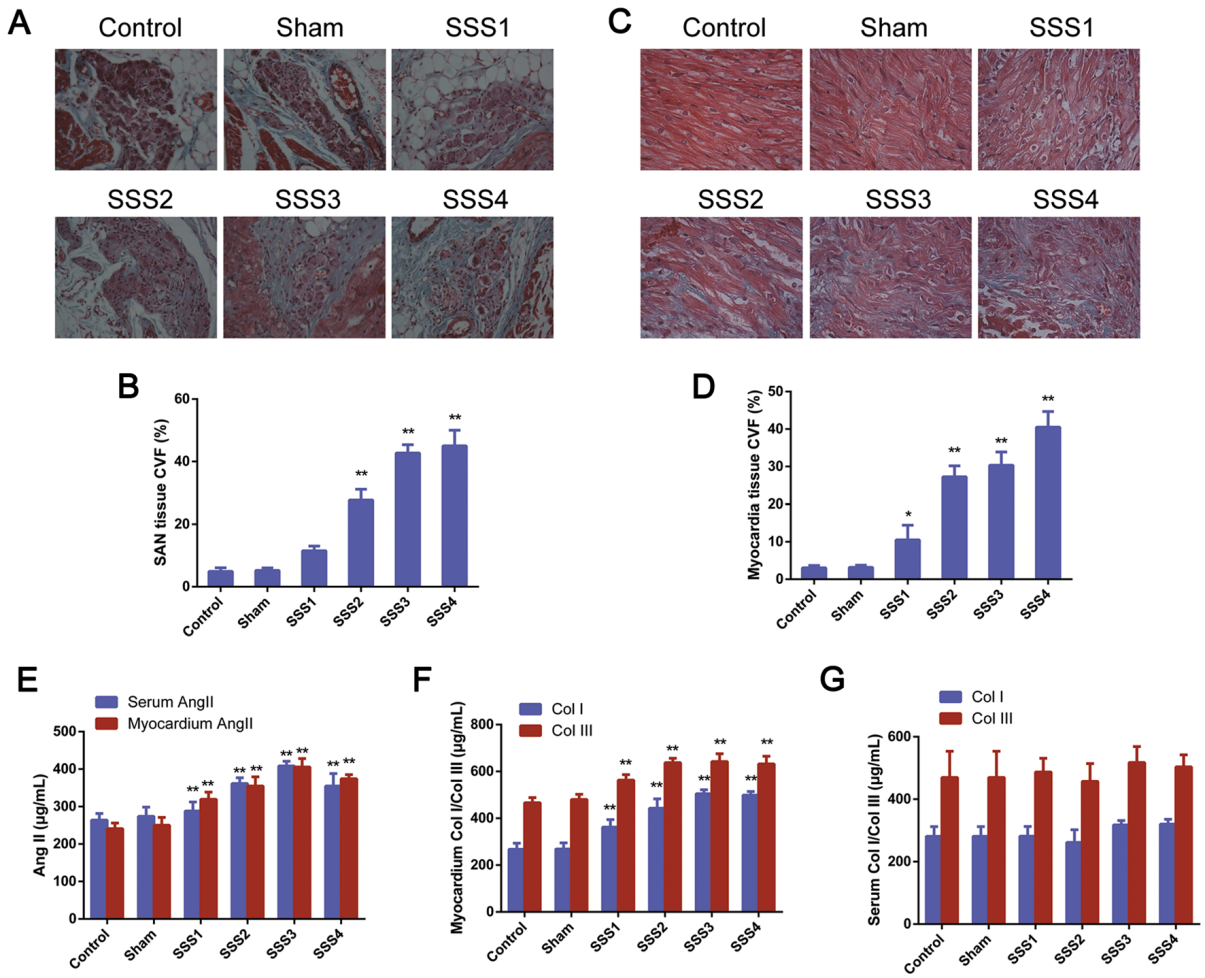
Increased SAN and atrial fibrosis and Ang II levels in SSS rats. **a** Representative images of Masson staining in SAN tissue. Ganglion cells of SAN tissues were stained in red and gaps between SAN tissues were stained in green with filamentous- and bundled shapes (scale bar 50 μm). **b** Statistical analysis of **a**. **c** Representative images of Masson staining of cardiomyocytes. Masson staining, red; collagen staining, green, with filamentous and bundled shapes (scale bar 25 μm). **d** Collagen volume fraction of myocardial tissues with Masson staining. Data are presented using the mean ± standard deviation (*n* = 8). *P < 0.05 and **P < 0.01 vs. control and sham group. **e** Increased Ang II levels in serum and SAN tissues of SSS rats. **f** Increased Col I and Col III levels in SAN tissues of SSS rats. **g** Increased Col I and Col III levels in the serum of SSS rats. All data are presented as the mean ± standard deviation (*n* = 8/group). **P < 0.01 vs. the control and sham group

We first evaluated the fibrosis in the SAN and atria of rats in six groups by Masson staining and found that the parenchyma cells in the SAN of the control and sham rats were neatly and densely arranged with clear structures and slight green staining in the intracellular space. However, the SSS1 rats exhibited disorganized parenchyma cells with clear connective tissues as well as more green staining in the intracellular space, which were more obvious in the SSS2, SSS3 and SSS4 rats (Fig. 2a). We also found that collagen levels in the SAN tissues of the SSS1 rats increased compared with those in the sham rats (Fig. 2b. *P *< 0.01), which was further increased in the SSS3 and SSS4 rats (Fig. 2b. *P *< 0.01 vs. SSS1). Consistent with the above observations, neat and well-organized myocardium with slight green staining by Masson staining in the control and sham rats, but disorganized myocardium with notable amounts of green-stained tissue between the myocardia in the SSS1 rats, and the disorganized myocardium and green staining were further increased in the SSS2, SSS3 and SSS4 rats (Fig. 2c). CVF analysis revealed increased myocardial collagen levels in SSS1 compared with that in the sham and control rats (Fig. 2d, *P *< 0.05), which were further elevated in the SSS2, SSS3 and SSS4 rats (*P *< 0.01). No significant difference in collagen levels was observed between the SSS3 and SSS4 groups (*P *> 0.05).

In addition, the serum Ang II and Ang II, Col I, and Col III levels in the SAN tissues in the SSS1 group were significantly higher than those in the sham group (*P *< 0.05), and SSS3 had the highest levels of serum or SAN Ang II among all four SSS groups, whereas no significant differences in Ang II, Col I, or Col III in the serum or SAN tissues were observed between the sham and control groups (*P *> 0.05, Fig. 2e–g).

### Increased expression of TRPM7 and p-Smad2/Smad2 ratio in the SAN of SSS rats

**Fig. 3:**
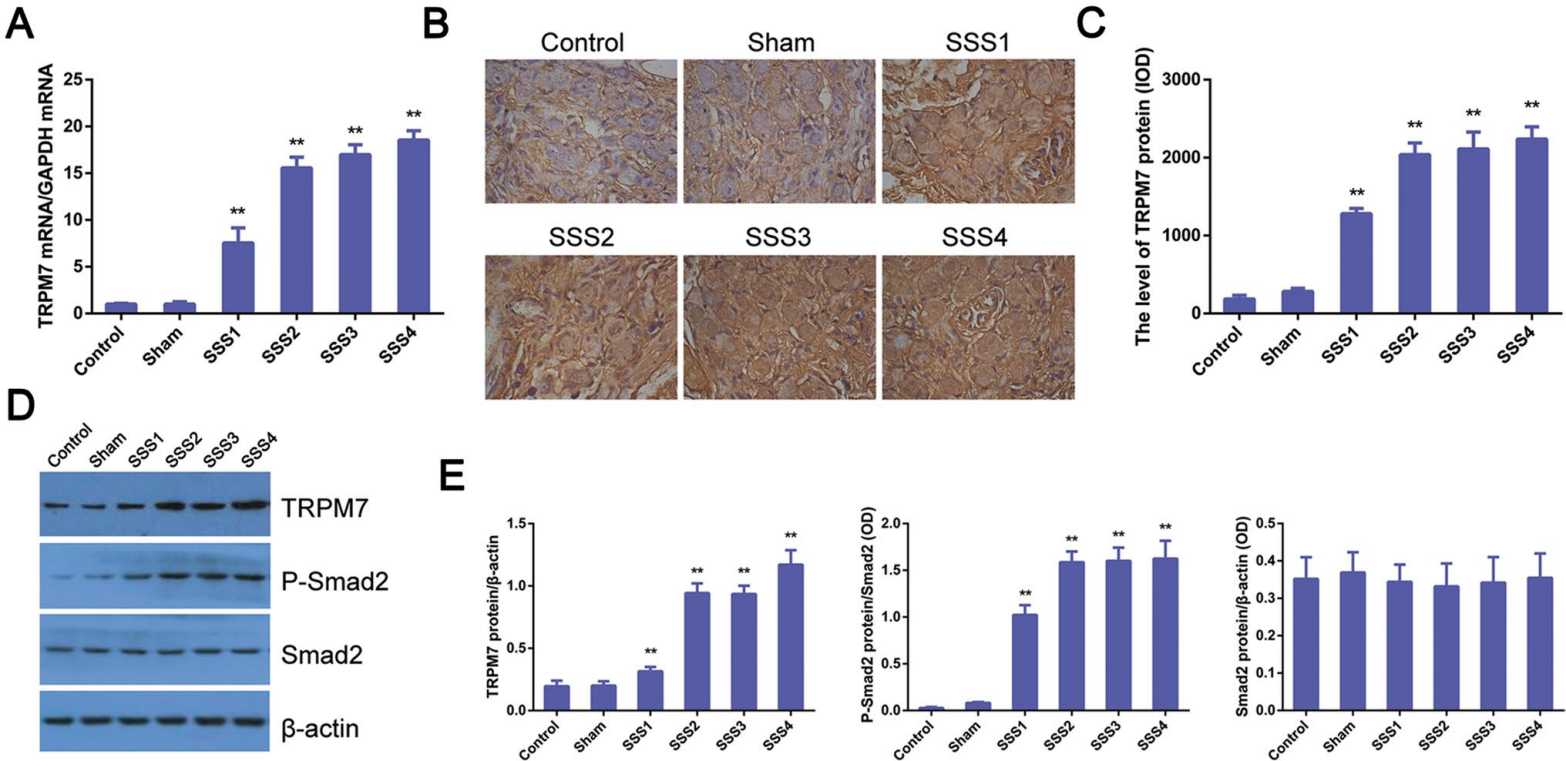
Increased levels of TRPM7 and p-Smad2 in SAN of SSS rats. **a** Increased TRPM7 mRNA expression in the right atria of SSS rats assessed by RT-qPCR. Data are presented as the mean ± standard deviation (*n* = 8). **P < 0.01 vs. control and sham group. **b** Representative images of TRMP7 immunohistochemistry in SAN tissues of control, sham and different SSS rats (scale bar 25 μm). **c** Quantitative analysis of **b**. The intensity of staining was measured as noted in “Materials and methods”. **d** Representative immunoblotting image showing increased expression of TRPM7 and pSmad2 in SSS rats. Western blot was performed as noted in “Materials and methods”. β-Actin was used as a loading control. **e** Statistical analysis of **d**. The gray levels of TRPM7 and Smad2 were normalized to b-actin, while the pSmad2 level was normalized to the total Smad2. All data are presented as the mean ± standard deviation (*n* = 8/group). **P < 0.01 vs. control and sham group

Next, we examined TRPM7 levels in the SAN and atrial tissues of rats of the six groups. Real-time PCR showed significantly elevated TRPM7 mRNA levels in the SSS1 group than in the control group (Fig. 3a. *P *< 0.01), which was further increased in the SSS2, SSS3, and SSS4 groups (*P *< 0.01), while no significant difference in TRPM7 mRNA expression was observed between the control and sham groups (*P *> 0.05, Fig. 3a). Additionally, TRPM7 immunohistochemistry showed a small amount of brownish yellow pigments in the intracellular space in SAN tissues of rats from the control and sham groups; however, a large amount of brownish yellow filament, small-strip, and flake-like was staining in the intracellular space of the rats in all SSS groups (Fig. 3b). Color intensity analysis of TRPM7 immunohistochemistry showed significantly higher TRPM7 expression in the SSS1 group than in the control group (Fig. 3c. *P *< 0.01), which was further elevated in the SSS2, SSS3, and SSS4 groups (*P *< 0.01). No significant difference in TRPM7 expression was observed between the SSS3 and SSS4 groups (*P *> 0.05, Fig. 3c). In line with the above findings, western blot revealed higher TRPM7 expression in all SSS groups than the control and sham groups (Fig. 3d, e, *P *< 0.01). We also found that p-Smad2 levels were higher in the SSS rats than the control and sham rats (*P *< 0.01), while the total Smad2 levels were comparable among these groups (*P *> 0.05, Fig. 3d, e).

### Ang II-induced levels of Col I and Col III and Smad2/p-Smad2 in CFs in vitro

**Fig. 4:**
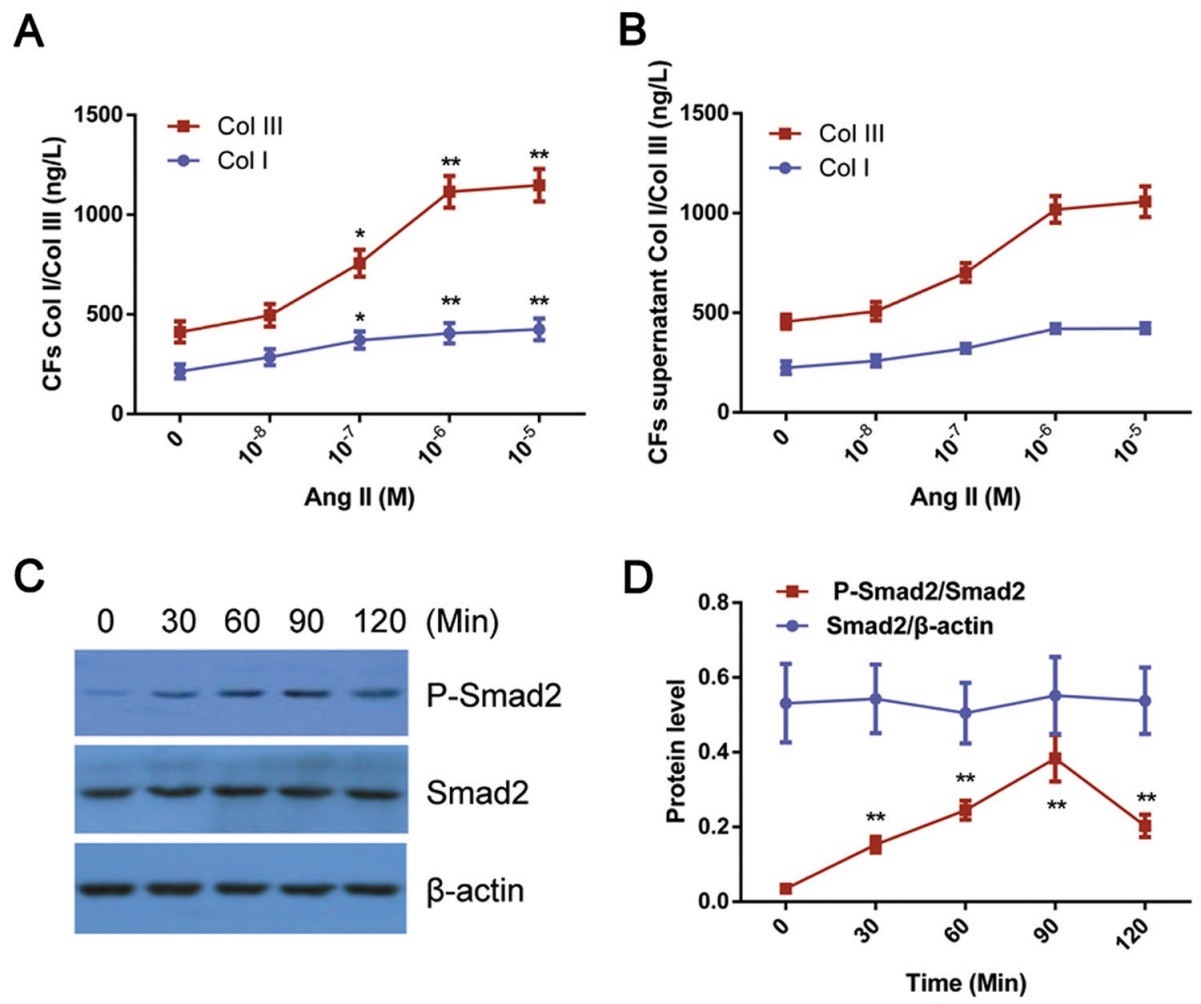
Ang II-induced levels of Col I and Col III and Smad2/p-Smad2 in CFs in vitro. **a**, **b** Increased Col I and III levels by Ang II treatment in CFs. Cultured CFs were stimulated with Ang II (10^6^ M) for 24 h followed by the measurement of Col I and III as described in “Materials and methods”. Data are presented as the mean ± standard deviation (*n* = 8/group). *P < 0.05 and **P < 0.01 vs. the Ang II 0 M group. **c** Ang II increased pSmad2 levels in CFs. Representative image of the immunoblot is shown. β-Actin served as a loading control. **d** Statistical analysis of **c**. All data are presented as the mean standard deviation (*n* = 3). **P < 0.01 vs. control; ^#^*P *< 0.05 vs. Ang II (10^−6^ M) treatment

We next examined whether Ang II could affect the expression of Col I and III and pSmad2 in CFs in vitro. As shown in Fig. 4a, b, Ang II (10^−8^–10^−5^ M) promoted Col I and Col III synthesis in CFs in a dose-dependent manner, and Ang II at 10^−6^ M increased and maintained Col I and Col III at the peak values (*P *< 0.01). Additionally, Ang II increased the p-Smad2 levels in CFs in a time-dependent manner as well and p-Smad2 was significantly elevated at 60 min and peaked at 90 min after Ang II treatment (10^−6^ M) (Fig. 4c, d).

### TRPM7 mediates the induction of p-Smad2 by Ang II in CFs in vitro

**Fig. 5:**
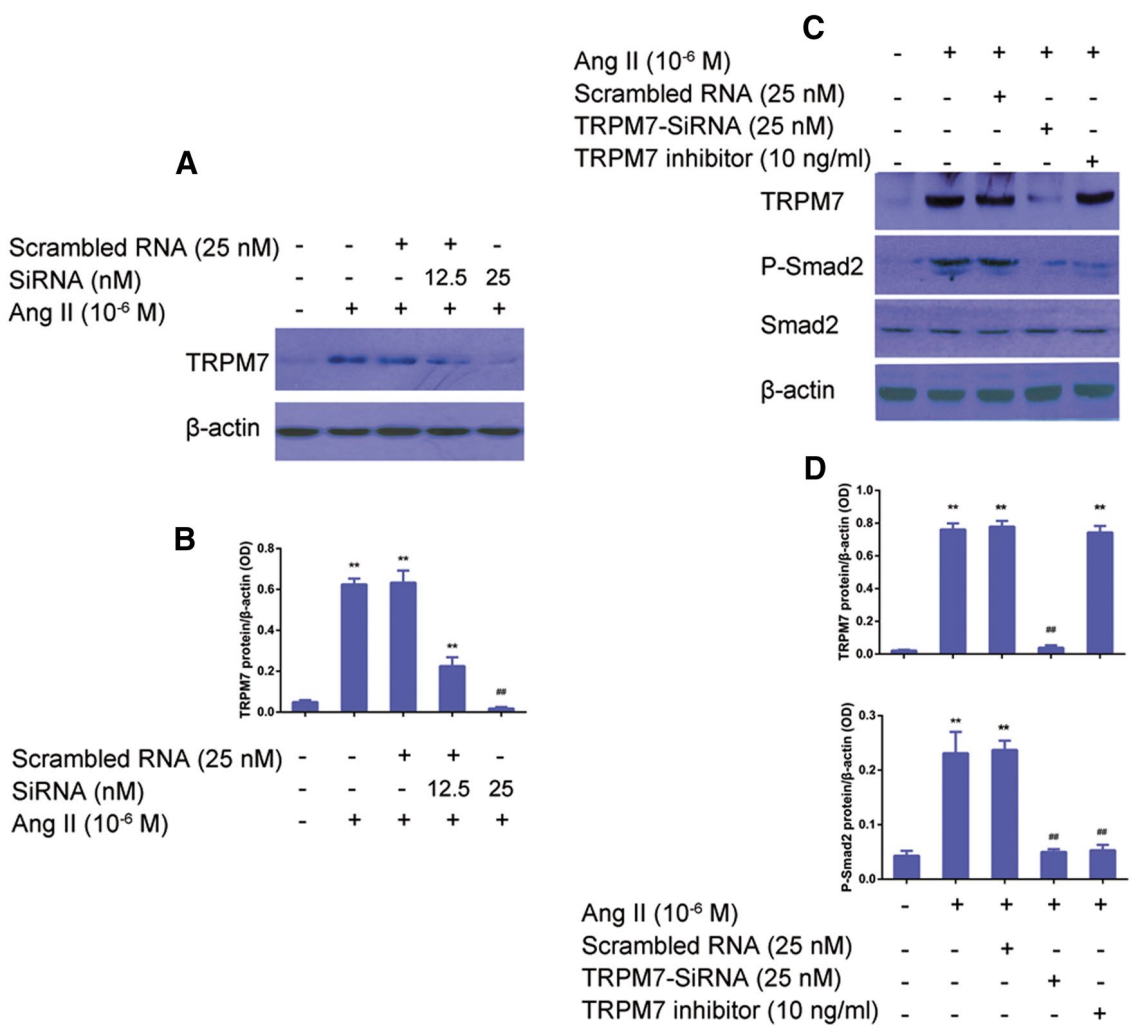
Knockdown of TRPM7 decreases Ang II-induced pSmad2 levels in CFs in vitro. **a** TRPM7 knockdown by siRNA suppressed the induction of TRPM7 by Ang II. A representative image of protein blot is shown. β-Actin was used as a loading control. **b** Statistical analysis of **a**. Data are presented as the mean ± standard deviation (*n* = 5). *P < 0.05 vs. the scrambled group; ^##^*P *< 0.01 vs. the 12.5 nM TRPM7-siRNA treatment group. **c** TRPM7 knockdown by siRNA suppressed the induction of pSmad2 by Ang II. A representative image of the protein blot is shown. β-Actin was used as a loading control. **d** Statistical analysis of **c**. Data are presented as the mean standard deviation (*n* = 5). *P < 0.05, vs. control; ^##^*P *< 0.01 vs. Ang II (10^−6^ M) treatment group or scrambled RNA (25 nM) + Ang II (10^−6^ M) treatment group

**Fig. 6:**
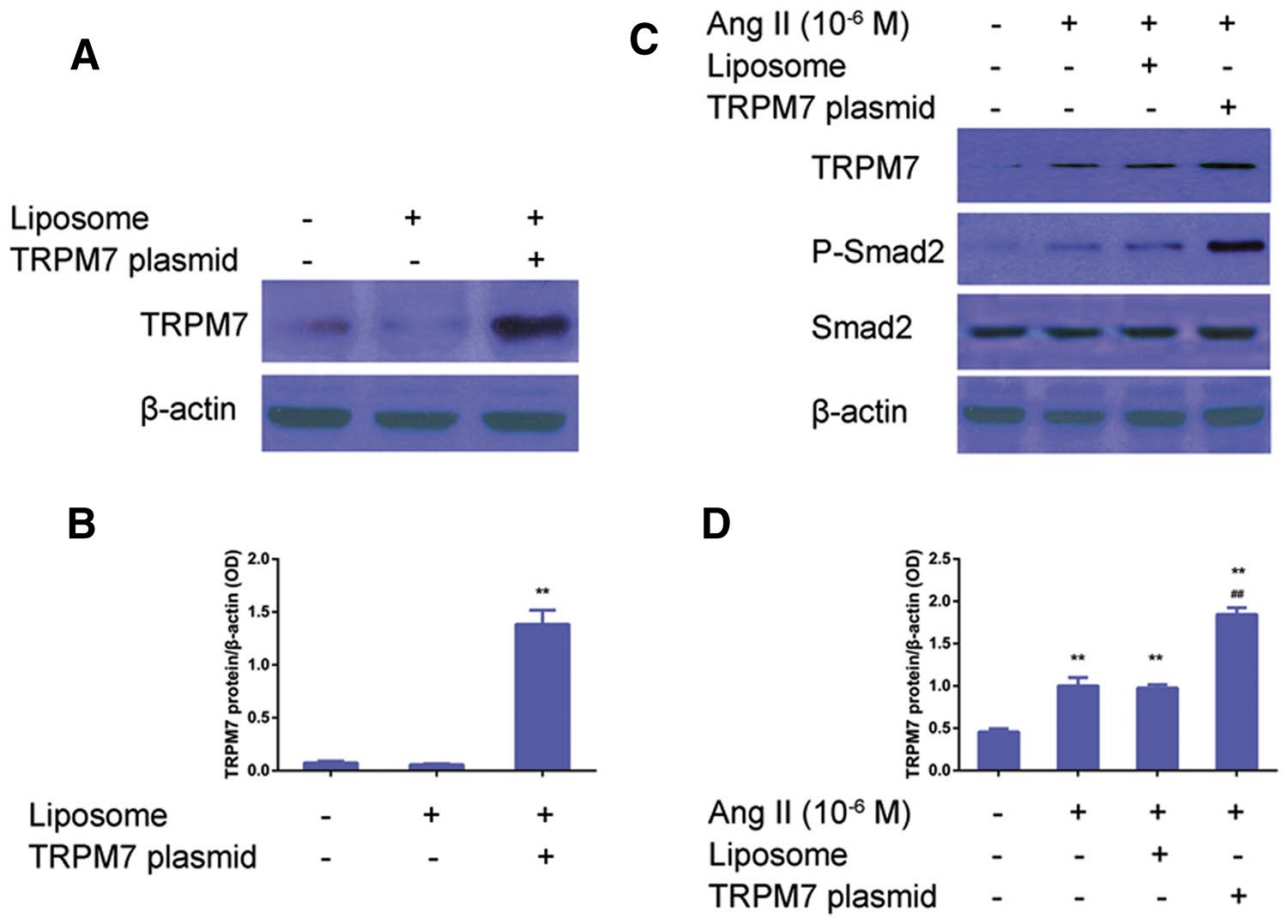
Overexpression of TRPM7 potentiates Ang II-induced pSmad2 levels in CFs in vitro. **a** Overexpression of TRPM7 in CFs. Transfection of TRPM7 encoding vector and western blot were performed as described in “Materials and methods”. A representative image of the immunoblot is shown. **b** Statistical analysis of A. Data are presented as the mean ± standard deviation (*n* = 5). **P < 0.01 vs. liposome alone group. **c** Overexpression of TRPM7 potentiated the Ang II-induced pSmad2 level. Western blot was carried out as described in “Materials and methods”. A representative image of the protein blot is shown. **d** Statistical analysis of **c**. Data are presented as the mean ± standard deviation (*n* = 5). **P < 0.01 vs. control; ^##^*P *< 0.01 vs. Ang II (10^−6^ M) treatment group or liposome + Ang II (10^−6^ M) treatment group

To investigate the role of TRPM7 in the regulation of Ang II-induced p-Smad2/Smad2 expression in CFs, TRPM7 siRNA was used to knock down TRPM7. CFs was transfected with either scrambled or TRPM siRNA and stimulated with Ang II (10^−6^ M) for 24 h. As shown in Fig. 5a, b, the TRPM7 expression in the CFs with Ang II treatment was higher than in the control CFs, which was significantly inhibited by TRPM7-siRNA (*P *< 0.05), while no significant difference in TRPM7 protein expression was found among the Ang II, the scramble RNA + Ang II, and the TRPM7 inhibitor groups (*P *> 0.05). It was found that TRPM7 siRNA inhibited Ang II -induced p-Smad2 expression in CFs (Fig. 5c, d); any significant difference in the total Smad2 expression between the groups was observed (*P *> 0.05). Then, a gain-of-function was used to examine the effects of TRPM7 on the Ang II-induced pSmad2 vs. Smad2 ratio. CFs transfected with TRPM7 encoding plasmid and treated with Ang II (10^−6^ M) for 24 h showed significantly higher levels of TRPM7 and p-Smad2 expression than in the control CFs (Fig. 6a, b). Correspondingly, CFs with Ang II (10^−6^ M) showed that the TRPM7 agonist significantly increased the p-Smad2 protein level, while no significant changes in the total Smad2 protein level were observed between these groups (Fig. 6c, d).

### TRPM7 mediates Ang II-induced Col I and Col III synthesis in CFs in vitro

**Fig. 7:**
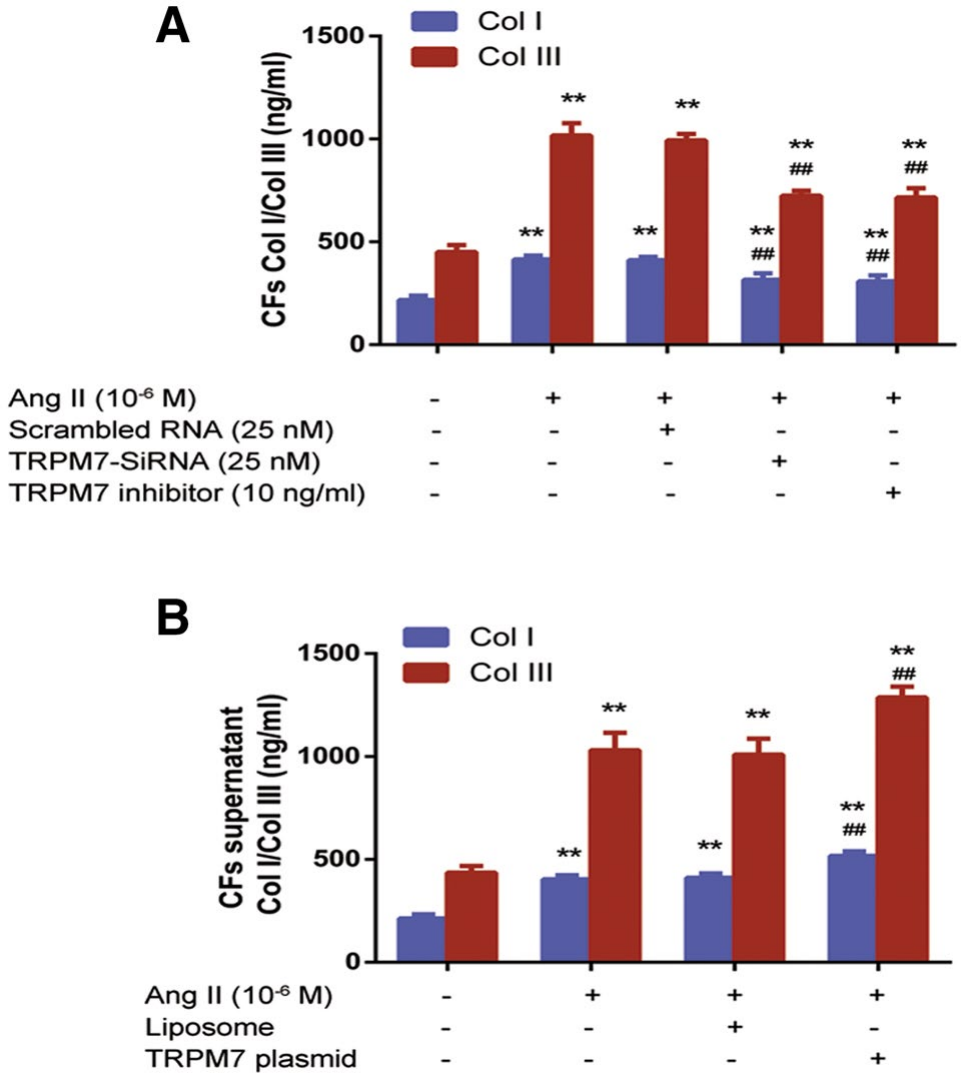
TRPM7 mediates Ang II-induced Col I and Col III synthesis in CFs in vitro. **a** Suppression of TRPM7 by siRNA or inhibitor decreased the Ang II-induced Col I and III levels in CFs. **b** Overexpression of TRPM7 increased the Ang II-induced Col I and III levels in CFs. ELISA was used to measure the Col I and III levels as described in “Materials and methods”. Data are presented as the mean ± standard deviation (*n* = 8). **P < 0.01 vs. control (blank control); and ^##^*P *< 0.01 vs. Ang II (10^−6^ M) treatment group

Similar loss- and gain-of-function approaches were used to evaluate the effects of TRPM7 knockdown on Ang II-induced Col I and Col III synthesis in CFs, and the Col I and Col III levels were assessed using ELISA. As shown in Fig. 7a, CFs treated with Ang II (10^−6^ M) had significantly higher Col I and Col III levels than vehicle-treated cells (*P *< 0.01) and no significant differences in Col I and Col III levels were found between the CFs treated with Ang II (10^−6^ M) and with scramble RNA (*P *> 0.05). CFs with transfected with TRPM7-siRNA had significantly lower Col I and Col III levels than vehicle-treated CFs (*P *< 0.01), while no significant differences in Col I and Col III levels were observed between CFs transfected with TRPM7-siRNA and CFs with TRPM7 inhibitor 2-APB (10 ng/ml) (*P *> 0.05). Correspondingly, the overexpression of TRPM7 further promoted the induction of Co I and III synthesis by Ang II (10^−6^ M) (*P *< 0.01, Fig. 7b).

## Discussion

The present study showed that Ang II activity was enhanced in SSS rats and that the Ang II/Smad2 signaling promoted collagen synthesis in the SAN tissues of SSS rats. We also found that TRPM7 regulated CFs proliferation and collagen synthesis of SAN tissues through this signaling pathway.

Studies of anatomical morphology have confirmed that the fiber tissues within SAN play an important role in maintaining normal pacing and conductive function and that pathological fibrosis within SAN affects SAN function, as evidenced by observations that a variety of bradyarrhythmias are associated with tissue-fibrosis-induced action potential generation and conduction [1]. The present study showed that collagen deposition of SAN tissues in SSS rats increased in a time-dependent manner and became significantly higher than in the control group, as revealed by increased levels of Col I and III in SAN tissues of SSS rats compared with that in the control and sham rats. These findings suggested that the incidence and development of myocardial fibrosis are closely related to the duration of SSS. This study also found that the increasing Ang II levels in the SAN tissues of SSS rats coincided with elevated TRPM7 and p-Smad2 expression, suggesting that CF activation may be regulated by Ang II/TRPM7/Smad2. Notably, a recent study showed that TRP and its mediated Ca^2+^-influx signal in the CFs of SSS patients play a key role in the transformation of CFs into CMFs [21]. In the present study, TRPM7 expression was increased in SAN tissues in SSS rats together with a significant increase in Smad2 phosphorylation level, suggesting the involvement of TRPM7/Smad2 in Ang II-mediated myocardial fibrosis. Many clinical studies have demonstrated that Ang II contributes to the cardiac remodeling that occurs in hypertensive heart disease, heart failure, myocardial infarction, cardiomyopathy, and paroxysmal atrial fibrillation with sick sinus syndrome [35–38]. In addition, Ang II expression is more pronounced in myocardial tissue with elevated cardiac load due to fibrosis than in normal myocardial tissues and is closely related to interstitial fibrosis in myocardium [39]. Mechanistically, Ang-II promotes fibrosis through activating TGFβ1 signaling; notably, in the absence of TGFβ1, Ang II failed to cause myocardial hypertrophy [40]. Smad2, a fibrosis factor and downstream factor of TGFβ1, is an important regulator of collagen synthesis in fibroblasts. In this study, Smad2 phosphorylation levels in SAN tissues were found to be significantly higher in SSS rats than in control rats, suggesting activated Smad2 signaling in the SAN tissues of SSS rats. Previous studies have shown that Ca^2+^ is an indispensable signal molecule participating in cell differentiation, gene expression, cell proliferation and growth, and cell death in various cells [41, 42] and that Ca^2+^ influx is essential to the biological functions in CFs during collagen synthesis [14]. In recent years, numerous studies have shown that TRPM7 mediates Ca^2+^ influx and thus plays a key role in the transformation of CFs into CMFs [43, 44]. Abnormal levels of TRPM7 were also potentially involved in fibrosis observed in patients with atrial fibrillation, as supported by the findings that the TRPM7 density on the CFs of these patients was upregulated three- to fivefold relative to healthy volunteers and its Ca^2+^ influx was also significantly increased [26]. Moreover, TRPM7 was shown to mediate Ca^2+^ and Mg^2+^ influx and thus takes part in Ang II-induced atrial fibrosis progression [22]. Thus, TRPM7 is widely believed to be involved in regulating the proliferation, differentiation, and collagen synthesis of CFs. The present study found that TRPM7 expression was upregulated in the SAN tissues and atrial muscle tissues in SSS rats and that prolonged progression of SSS further increased TRPM7 expression, suggesting that elevated levels of TRPM7 may be closely correlated with the cardiac fibrosis that occurred in SSS rats.

Our in vitro experiments showed that Ang II stimulated Col I and Col III synthesis in CFs in a time- and dose-dependent manner, suggesting the presence of an Ang II-induced signaling pathway in CFs. A previous study reported that Ang II binds to AT1 receptor to activate the PKC pathway, leading to an increase in intracellular Ca^2+^ concentration and thus mediating a series of biological effects, such as cell proliferation, while intracellular Ca^2+^ concentration was closely associated with collagen fiber synthesis in CFs [45]. Here, we showed that Ang II stimulated CFs and increased the levels of TRPM7 and pSmad2. To confirm the relationship between Ang II, TRPM7, and Smad2, we first detected a significant elevation of p-Smad2 protein levels in CFs after Ang II stimulation, suggesting that Ang II/Smad2 is one of the signaling pathways that contributes to collagen synthesis in CFs. We then used loss- and gain-of-function approaches to examine the involvement of TPRM7 in Ang II-mediated increase in pSmad2 levels. The knockdown of TPRM7 decreased but overexpression of TPRM7 increased the pSmad2 levels induced by Ang II stimulation. Moreover, the TPRM7 inhibitor, 2-APB, showed similar effects as those of TPRM7 siRNA on Smad levels. Taken together, these results indicated that TRPM7 was an important molecule involved in regulating Ang II-promoted Smad2 synthesis and fibrosis.

The present work has several limitations. For instance, the TRPM7 channel protein is a bifunctional protein with ion channel and protein kinase structures; however, we only studied its protein kinase activity and did not use electrophysiological techniques to investigate its ion channel properties. Further studies will be necessary to determine if these two functions act independently or interact with each other. Also, many signaling pathways are involved in myocardial fibrosis, but we only studied the effect of TRPM7 on collagen synthesis in the myocardium through Ang II/Smad2 signaling. Further study will be needed to determine if TRPM7 plays a regulatory role in other myocardial fibrosis signaling pathways. In addition, because there are many TRP channel protein superfamily members present in CFs, further investigation of other TRP channel protein superfamily members and their involvement in regulating the formation of myocardial fibrosis in SSS rats will be needed.

## Conclusions

We demonstrate that Ang II activity is enhanced in SSS rats and that it involves Ang II/Smad2 signaling, which promotes collagen synthesis at least in part through TRPM7. Thus, our findings suggest that the activation of TRPM7/Smad2 signaling by Ang II is an important mechanism leading to myocardial fibrosis in SAN tissues in SSS rats, indicating that TRPM7 is a potentially therapeutic target for SSS treatment.

## Abbreviation

Ang II: Angiotensin II
CFs: Cardiac fibroblasts
CMFs: Cardiac myofibroblasts
SAN: Sinoatrial node
SD: Sprague–Dawley
SSS: Sinus sick syndrome
TRP: Transient receptor potential
TRPM: Transient receptor potential melastatin
TRPM7: Transient receptor potential subfamily M member 7
